# Tracheobronchial reconstruction by inverted Barclay’s method for tracheobronchial injury in an 8-year-old girl: a case report

**DOI:** 10.1186/s40792-022-01405-w

**Published:** 2022-03-28

**Authors:** Yamato Suzuki, Hisato Ishizawa, Hiroshi Kawai, Yasushi Matsuda, Yasushi Hoshikawa

**Affiliations:** Department of Thoracic Surgery, Fujita Health University School of Medicine, 1-98 Dengakugakubo, Kutsukake-cho, Toyoake, Aichi 470-1192 Japan

**Keywords:** Child, Tracheobronchial injury, Surgery, Reconstruction

## Abstract

**Background:**

Tracheobronchial injury in children is rare but can be highly fatal in severe cases. Therefore, prompt diagnosis and treatment are required. The appropriate treatment method depends on the extent and severity of the injury.

**Case presentation:**

An 8-year-old girl fell from the fifth floor and was transported to a local hospital. She had a tracheobronchial injury, went into cardiopulmonary arrest during transportation to our hospital. She was revived with cardiopulmonary resuscitation, and veno-arterial extracorporeal membrane oxygenation (VA-ECMO) was commenced. Subsequently, we performed tracheobronchial reconstruction by inverted Barclay’s method for tracheobronchial injury. She was switched from VA-ECMO to venovenous (VV)-ECMO 4 days postoperatively, and VV-ECMO was eventually discontinued 27 days after the surgery. The patient was awake and weaned off the ventilator on postoperative day 58. She was discharged 97 days after the surgery.

**Conclusions:**

Tracheobronchial reconstruction by inverted Barclay’s method is the preferred surgical technique when other reconstruction techniques are expected to cause excessive tension on the anastomosis of the right main bronchus.

## Background

Tracheal and bronchial injuries due to chest trauma in children are uncommon, but the fatality rate is reported to be 30%, with approximately half of those deaths occurring within the first hour after injury [1]. Therefore, prompt diagnosis and treatment are necessary. Treatment is determined based on the extent and severity of the injury [2]. Direct suturing and pericardial coverage of the injured area have been reported [3–6]. We present a case of an 8-year-old girl who survived a traumatic tracheobronchial injury after undergoing tracheobronchial reconstruction by inverted Barclay’s method.

## Case presentation

**Fig. 1:**
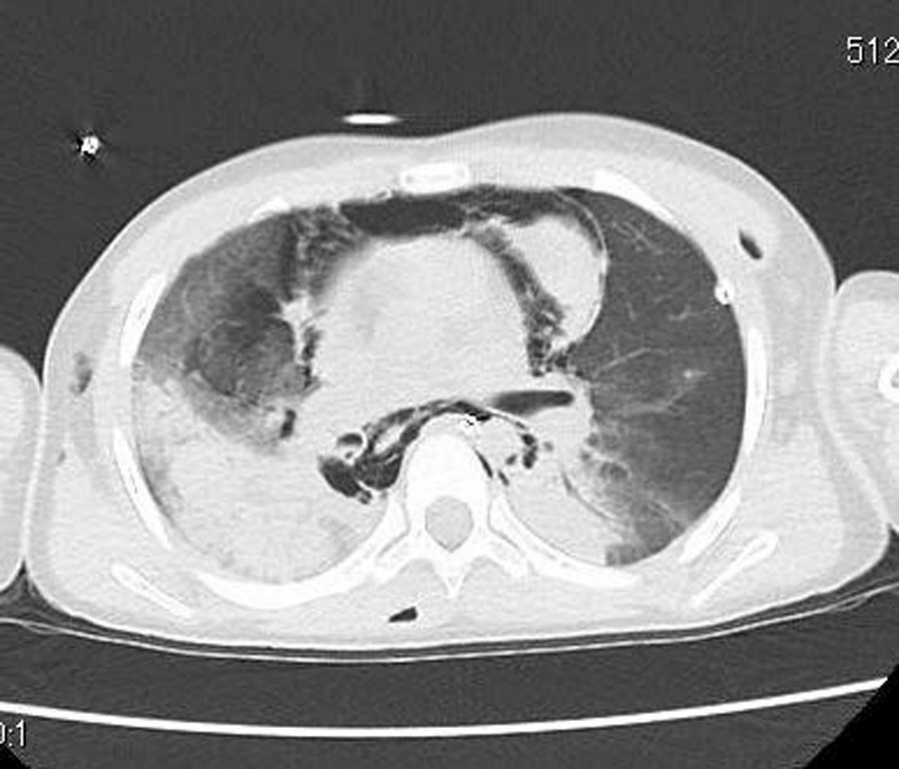
Computed tomography image obtained before surgery showing injury of the tracheal bifurcation, and the right and left main bronchi

**Fig. 2:**
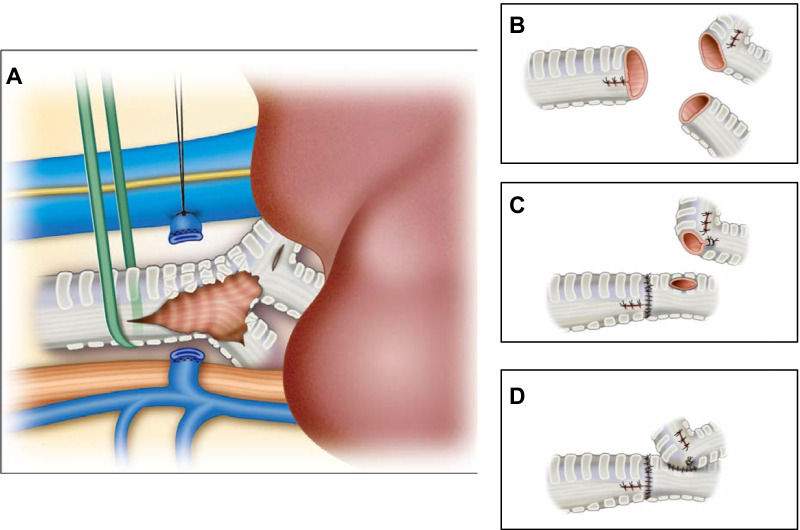
Schematic overview. **A** Injured area of the tracheal bifurcation. **B** Resection of the injured area and suturing of the laceration area. **C** Anastomosis of the trachea and left main bronchus. **D** Anastomosis of the left and right main bronchi

**Fig. 3:**
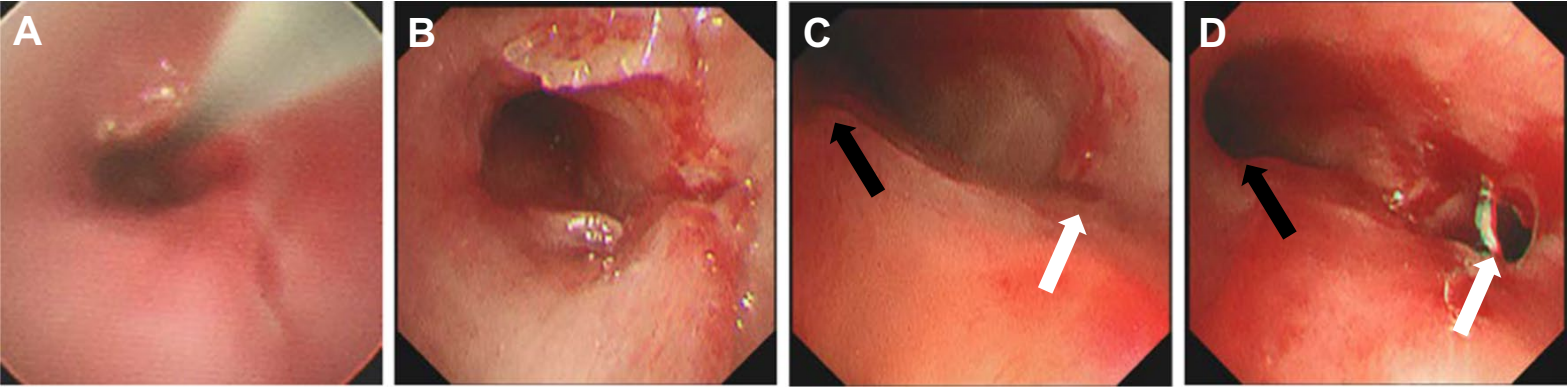
Bronchoscopic findings of anastomosis. **A**, **B** Stenosis of the anastomosis between the trachea and left main bronchus. **A** Before treatment, with wires for balloon dilation. **B** After treatment. **C**, **D** Stenosis of the anastomosis between the left main bronchus (black arrow) and right main bronchus (white arrow). **C** Before treatment. **D** After treatment

An 8-year-old girl fell from the fifth floor and was transported to a local hospital. She had a tracheobronchial injury (Fig. 1), bilateral hemopneumothorax, traumatic subarachnoid hemorrhage, liver injury, and fracture of the left fibula. She experienced cardiopulmonary arrest during transportation to our hospital and underwent a successful cardiopulmonary resuscitation (CPR). On arrival at our hospital, we inserted a drain into the right thoracic cavity and transferred her to the operating room, where she experienced another cardiopulmonary arrest. We performed CPR and introduced veno-arterial extracorporeal membrane oxygenation (VA-ECMO). After she was revived and regained normal cardiac output, surgery was performed. A posterolateral skin incision was made, and the right thorax was opened. The tracheal bifurcation membrane was missing, and the cartilage in the area was injured. Furthermore, there were membrane lacerations on the cephalic side of the trachea and right upper bronchus (Fig. 2A). The right lung was tense and congested. We resected the tracheal bifurcation, two cephalic rings of the tracheal cartilage, two rings of the right main bronchus, and one ring of the left main bronchus. We closed the membrane laceration of the trachea and right upper bronchus with interrupted sutures using 4-0 PDS sutures (Ethicon, USA; Fig. 2B). We dissected the right pulmonary hilum and made a U-shaped pericardial incision around the pulmonary vein for pericardial release. In addition, we performed tracheobronchial reconstruction by inverted Barclay’s method because a little movement of the right main bronchus was observed. The trachea and left main bronchus were reconstructed using 4-0 PDS sutures with interrupted sutures in the cartilaginous portion and running sutures in the membranous portion (Fig. 2C). A foramen was created in the left main bronchus and anastomosed to the right main bronchus using 4-0 PDS sutures with interrupted sutures for the cartilage and running sutures for the membranous portion (Fig. 2D). VA-ECMO was continued, and the patient was admitted to the intensive care unit (ICU). On postoperative day 1, the anastomosis was patent, but complete bilateral atelectasis persisted. We switched from VA-ECMO to venovenous (VV)-ECMO 4 days post-surgery. On postoperative day 12, we performed another surgery on the right hemothorax. VV-ECMO was discontinued 27 days after the surgery. The patient was awake and weaned off the ventilator 58 days postoperatively. She was discharged from the ICU 64 days after the surgical procedure. Bronchoscopy showed anastomotic obstruction by granulation because of perioperative ischemia. On postoperative day 85, the granulation was removed, and balloon dilation was performed (Fig. 3). Although the patient had a minor short-term memory impairment and needed some support because of the difficulty in moving her left foot due to the left fibula fracture, she was discharged 97 days after the surgery walking. After discharge, she went to a public normal elementary school.

## Discussion

Reports have described direct suturing of a tracheobronchial injury in children [3–5]. In those cases, the injured areas were narrower than those in our case. In our case, the membranous area defect was so extensive that direct suturing was not possible. In another report, the pericardium was used to repair a tracheobronchial injury in a child [6]. However, in that case, suture failure occurred after surgery, requiring reinforcement of the sutures with the latissimus dorsi muscle. Insufficient blood flow and bacterial contamination of the injured area may have caused suture failure. The injured area, in that case, was similar to that in our case. Pericardial closure may have been an option in our case; however, there was a risk of suture failure owing to the reasons described above. Therefore, we did not choose that method.

Recently, it has been reported that covered self-expanding metallic stent (SEMS) implantation can be used to treat tracheobronchial injuries with high surgical risk [2]. Treatment of such injuries with a covered SEMS involves two processes. First, the SEMS covers the defect and serves to clear the airway. Second, prolonged implantation allows inflammatory cells to infiltrate the defect, causing granulation and healing. It is necessary to adjust the size and shape of the SEMS to fit the injured area. In our case, the patient’s circulation was unstable, the airway needed urgent reconstruction, and there was little time to prepare a SEMS that would fit. Even if a suitable SEMS had been available, the extent of the membranous defect was so large that stenting would not have resulted in granulation.

If direct suturing, pericardial patch, and covered SEMS are not suitable treatments for a tracheobronchial injury, reconstruction of the injured area is necessary. Before performing reconstruction, resection of the injured area is necessary, as reconstruction without resection may lead to postoperative suture failure and anastomotic stenosis owing to insufficient blood flow and bacterial contamination. Given these risks, we resected the injured area, though the injury was extensive. Reconstruction methods for tracheal bifurcation include the double-barreled, Barclay’s, inverted Barclay’s, and Grillo’s methods [7–10]. Tension-free anastomosis should be selected because excessive tension on the anastomosis can cause suture failure. In our case, the right main bronchus had a limited range of motion that may have been caused by pulmonary congestion. Double-barreled, Barclay’s, and Grillo’s methods might have led to excessive tension on the anastomosis, resulting in suture failure. Therefore, we chose an inverted Barclay’s method, in which the anastomosis of the right main bronchus was formed caudally. As a result, suture failure did not occur. Although stenosis due to defective granulation of the right main bronchus occurred once, we performed bronchoscopic curettage of the granulation followed by balloon dilation, and no restenosis was observed afterward.

## Conclusions

This report described an 8-year-old girl who survived a tracheobronchial injury after resection of the injured area and tracheobronchial reconstruction by inverted Barclay’s method. This reconstruction method may be effective when other methods are more likely to cause excessive tension on the anastomosis of the right main bronchus.

## Data Availability

The data in the current study are available from the corresponding author on request.

## Abbreviations

CPR: Cardiopulmonary resuscitation
VA-ECMO: Veno-arterial extracorporeal membrane oxygenation
ICU: Intensive care unit
VV-ECMO: Venovenous extracorporeal membrane oxygenation
SEMS: Self-expanding metallic stent
